# Evidence for Vocal Learning and Limited Dispersal as Dual Mechanisms for Dialect Maintenance in a Parrot

**DOI:** 10.1371/journal.pone.0048667

**Published:** 2012-11-06

**Authors:** Alejandro Salinas-Melgoza, Timothy F. Wright

**Affiliations:** Department of Biology, New Mexico State University, Las Cruces, New Mexico, United States of America; Utrecht University, The Netherlands

## Abstract

Studies of avian vocal dialects commonly find evidence of geographic and acoustic stability in the face of substantial gene flow between dialects. The vocal imitation and reduced dispersal hypotheses are alternatives to explain this mismatch between vocal and genetic variation. We experimentally simulated dispersal in the yellow-naped amazon (*Amazona auropalliata*) by moving individuals within and across dialect boundaries in Costa Rica. One juvenile translocated across dialect boundaries altered its contact call to imitate the acoustic form of the local call six weeks post-release. In contrast, four adults translocated across dialect boundaries returned to their original capture site within 120 days, while five cross-dialect translocated adults who remained at the release site did not alter their contact calls. Translocated individuals were observed to show some segregation from resident flocks. The observation of vocal imitation by the juvenile bird supports the vocal imitation, whereas the behavior of adults is more consistent with the reduced dispersal hypotheses. Taken together, our results suggest that both post-dispersal learning by juveniles and high philopatry in adults could explain the stability of vocal dialects in the face of immigration and gene flow.

## Introduction

Vocal dialects are a common manifestation of vocal learning, in which variation in calls or songs is much lower within than between geographic regions. Vocal dialects in birds were first described in white-crowned sparrows, *Zonotrichia leucophrys nutalli* [1], and subsequently reported in many other species [2]. Although cultural evolution theory predicts that dialects may change through learning errors and introduction of new call variants by immigrants [3], evidence of long-term stability of geographic boundaries and acoustic structure has been found for a number of species [4], [5], [6]. The forces promoting this stability, however, remain unclear.

Two main hypotheses have been proposed for the long-term maintenance of vocal dialects. The reduced dispersal hypothesis proposes that individuals rarely immigrate across dialect lines because they encounter fitness costs when associating with individuals using different acoustic signals. A reduction in fitness could result from assortative mating or social association by call type by the immigrants in the new dialect [1], [7]. Conversely, the vocal imitation hypothesis states that immigrants imitate the local call type upon arrival to a new dialect to facilitate interactions with new social groups [7], [8]. These hypotheses predict different patterns of a) post-dispersal vocal learning, b) genetic differentiation between dialects, c) cross-dialect dispersal, and d) fitness costs during social integration [7]. The reduced dispersal hypothesis predicts limited dispersal across dialect lines relative to within dialects, limited vocal learning by immigrants, increased fitness costs for immigrants during social integration due to differences in call type, and genetic differentiation among dialects. The vocal imitation hypothesis predicts the converse, namely that cross-dialect immigration will occur, immigrants will show vocal imitation post-dispersal, limited fitness costs for immigrants during social integration, and genetic homogenization of dialects.

Current evidence provides mixed support for the two hypotheses. Studies of mating preferences using individuals from different dialects typically find a stronger positive response between same dialect individuals [9], [10], suggesting that individuals with a different song or call type may suffer fitness costs relative to individuals using the local dialect (but see [11]). In contrast, population genetic studies typically indicate genetic differentiation among dialects is either weak or entirely absent, suggesting frequent dispersal across dialects boundaries [12], [13], [14], [15], [16], [17]. To date, however, there is little direct evidence of post-dispersal vocal learning by immigrants. What evidence exists is mainly from individuals that were non-systematically monitored and imitate the local call type after shifting territories post-dispersal [18], [19]. Empirical studies on cross-dialect dispersal patterns based on mark-recaptures are inconsistent; some indicate extensive movement of individuals across dialect boundaries [20], while others suggest such movement is limited [21]. Furthermore, controversy in the identification of the actual place of origin of marked individuals in one of those studies [21] reduces the contribution of this evidence to the reduced dispersal hypothesis [13].

The yellow-naped amazon (*Amazona auropalliata*) is a medium size parrot that exhibits three distinct vocal dialects in Costa Rica with well defined boundaries [22]. As in other dialect systems, there is evidence of temporal and spatial stability [5], stronger response to same dialect breeding territory intrusions [10], and indirect genetic evidence for movement of individuals across dialects [12], [16]. We experimentally simulated dispersal of wild yellow-naped amazons across vocal dialects to evaluate the relative importance of different mechanisms for dialect maintenance. If vocal imitation were the primary factor influencing dialect stability in this population, we predicted that experimentally translocated parrots would imitate the local calls upon introduction into another dialect area. Conversely, if reduced dispersal was the primary factor influencing dialects stability, we predicted that translocated individuals would not learn the new call type and would experience greater social segregation from local groups. As a control, we also translocated birds within the same dialect and observed their movement patterns.

## Methods

### Ethical Statement

This study was followed ethical animal treatment guidelines and was conducted in accordance with current regulations in Costa Rica and the United States. Research was conducted under permits ACG-PI-12-2006, ACG-PI-006-2007, ACG-PI-019-2008, and ACG-PI-010-2009 granted by the Costa Rican government and the Institutional Animal Care and Use Committee of New Mexico State University approval (protocol 2006-027) and with permission from the land owners of our study sites.

### Trapping and Translocation

**Figure 1:**
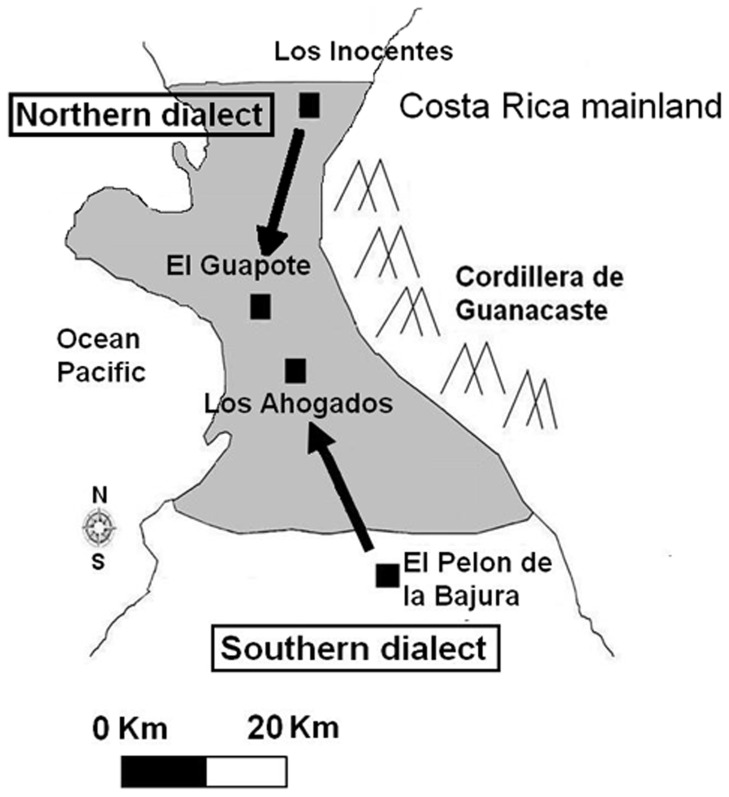
Map of the study area depicting vocal dialects, roosting sites (black solid squares), and movement of translocated individuals (arrows).

We captured parrots in May to August from 2006–2008 and from January to May in 2009 using canopy mist-nets in trees adjacent to four roosts in privately owned lands (Fig. 1). Individuals were captured using local contact calls and duets to attract them to canopy mist nets adjacent to roosts. In four cases, two individuals were captured at the same time, the first case was two individuals translocated within the Northern dialect in 2007, the second case corresponded to individuals translocated across dialect lines in 2008 and the final two of these cases were individuals that were released at the capture site in Los Ahogados in 2008. These captures raised the possibility that individuals were socially or genetically related; however, due to the high densities of birds at roosts and lack of individual markings, we were not able to identify the preexisting relationships among captured individuals. We fitted SI-2C, Holohil Systems neck-collar radio-transmitters to 24 adults and two juvenile from the Northern dialect and 13 adults and two juveniles from the Southern dialect. This unbalanced representation of age classes likely reflects the impact of rampant nest poaching in the area [23], which leads to low recruitment of new individuals. Individuals captured in the Los Ahogados (n = 18, Northern dialect) roost were released at the same site after being radio-collared as controls for capture and handling. Individuals from the El Pelon de la Bajura (n = 15, Southern dialect) and Los Inocentes (n = 8, Northern dialect) sites were transported approximately 30 km to Los Ahogados and El Guapote roosts (Northern dialects) for release as cross-dialect or within-dialect translocations (Fig. 1). Although typical dispersal distances are unknown for this species, the distance of 30 km that we moved birds is considerably greater than the typical daily foraging movements we recorded from non-translcoated birds at either El Pelon de la Bajura (834±45 m) or Los Ahogados (3978±205 m)(A Salinas-Melgoza and T.F. Wright unp. data). Captured individuals from both sites were alternated between these two recipient sites, one individuals being released in Los Ahogados and the next captured individual being released 13 km away at El Guapote site so as to better simulate long-distance dispersal by a single individual into a new dialect.

### Monitoring of Behavior and Vocalizations

We located radio-collared individuals using directional antennas and approached them within 50 m for observations. We attempted to obtain behavioral observations and vocal recordings from radio-collared individuals at least four times a week after release. Due to the wide-ranging movements of this species, not every individual was located during all periods. When a radio-collared individual was located, we alternated five minute focal observations on the radio-collared individual and on an unmarked individual in the same flock. We recorded every instance of aggressive acts (displacement and attack) or affiliative acts (allopreening and play) directed towards all translocated birds. To obtain an estimate of each individual’s original call structure, we recorded radio-collared birds’ contact calls immediately after release while individuals were flying, foraging or socializing with a Sennheiser ME67 shotgun microphone on a Marantz PMD670 or PMD660 solid state recorder. We continued to obtain vocal recordings throughout the study period to quantify change in vocal structure over time by recording individuals whenever located.

### Analysis of Vocal Change

To analyze vocal change in translocated individuals, vocal samples were divided *a priori* into a first phase (<6 weeks post-release) and a second phase (>6 weeks post-release). Six of the cross-dialect translocated individuals from the Southern dialect fulfilled the minimum requirements of being tracked for >6 weeks and having at least one good quality recording bout per phase (four individuals from 2007 and two from 2008, from which one was a juvenile from 2008 and five were adults from 2007and 2008; see Table 1). Seven out of the eight within-dialect translocated individuals from the Los Inocentes site spent most of the post-release period in mountainous terrain out of tracking range, resulting in very few recordings, observations, or locations; these individuals were excluded from further vocal and social analysis (Table 1).

**Table 1:** Summary of captured yellow-naped amazons indicating analysis in which they were included.

ID #	Year	Dialect oforigin	Treatment	Tracking days	Returned to capture site	Vocal imitation	Analysis performed	
							Distance to roost	Behavioral acts
0801Ŧ	2008	Southern	Cross-dialect	115	No	Yes	Yes	Yes
0703	2007	Southern	Cross-dialect	78	Yes	No	Yes	Yes
0705	2007	Southern	Cross-dialect	76	Yes	No	Yes	Yes
0706	2007	Southern	Cross-dialect	67	Unknown	No	Yes	Yes
0707	2007	Southern	Cross-dialect	56	Unknown	No	Yes	Yes
0808	2008	Southern	Cross-dialect	48	Unknown	No	Yes	
0602Ŧ	2006	Northern	Same roost	50			Yes	
0605	2006	Northern	Same roost	23			Yes	
0606	2006	Northern	Same roost	23			Yes	
0607	2006	Northern	Same roost	22			Yes	
0608	2006	Northern	Same roost	20			Yes	
0819	2008	Northern	Same Roost	75			Yes	Yes
0708Ŧ	2007	Southern	Cross-dialect	60	Unknown		Yes	Yes
0813	2008	Southern	Cross-dialect	30	Unknown			Yes
0711	2007	Northern	Within dialect	43	Yes			Yes
0713	2007	Northern	Within dialect	42	Yes			Yes
0814	2008	Southern	Cross-dialect	3	Yes			Yes
0816	2008	Northern	Same roost	24				Yes
0710	2007	Northern	Within dialect	5	Yes			
0806	2008	Southern	Cross-dialect	26	Yes			
0807	2008	Northern	Within dialect	36	Yes			
0913	2009	Northern	Within dialect	76	No			
0802	2008	Northern	Within dialect	7	Unknown			
0803	2008	Northern	Within dialect	21	Unknown			
0912Ŧ	2009	Northern	Within dialect	1	Unknown			
0701	2007	Southern	Cross-dialect	14	Unknown			
0702	2007	Southern	Cross-dialect	3	Unknown			
0805	2008	Southern	Cross-dialect	44	Unknown			
0809	2008	Southern	Cross-dialect	5	Unknown			
0820	2008	Southern	Cross-dialect	4	Unknown			

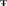
Denotes juveniles.

Only individuals included in the vocal imitation analysis are indicated with its respective output. Although some cross-dialect individuals were not included in any analyses (marked as unknown in the return to capture site column), they were included in this table to provide a complete view of the fate of translocated individuals.

We performed two analyses to assess vocal imitation. We selected 5–10 calls with good signal-to-noise ratio for each of these six translocated individuals per phase, and also for five breeding birds previously recorded in 2005 from Los Ahogados area in the Northern dialect (Fig. 1). We first performed spectral contour cross-correlations on these calls using the CORMAT command in Signal 5 (Engineering Design). We used spectrograms with a 256 sample FFT, band-passed filtering from 0.6 to 5.0 kHz, time normalization, and frequency shifting. We averaged the peak cross-correlation values within each individual-by-individual comparison and performed a principal coordinates analysis (PCO) on this similarity matrix using XLSTAT. Changes in similarity of calls between phases were explored by plotting the first two PCO eigenvectors. Second, we quantified 11 time and frequency parameters of the same calls [5]. We obtained three time (first and second segment duration, and total note duration) and eight frequency (first and second segment high frequency, first and second segment low frequency, first and second segment frequency range, first and second segment peak frequency) parameters [5] from spectrograms using onscreen cursors and automated procedures in Raven 1.4 (Cornell Laboratory of Ornithology). We performed discriminant function analysis with SPSS using these 11 parameters to validate a visual and auditory classification of the calls.

### Analysis of Social Integration

We evaluated the process of social integration of across-dialect translocated individuals to the resident flocks in three ways. First, we determined the degree of segregation of translocated individuals, which could provide an indication of the social costs individuals were incurring according to call type. We recorded the proportion of diurnal sightings per week a translocated individual was recorded alone, with resident birds only, with translocated birds only or with both type of birds in the flock. Secondly, as traditional roosts are important congregation sites [5], we determined whether translocated individuals were capable of tracking the location of these socializing sites in the recipient site. We obtained the distance from roosting locations to the closest main traditional roosts in the study area for six resident (five from 2006 and 1 from 2008) and seven across-dialect translocated individuals (five from year 2007 and two from year 2008, see Table 1). Finally, we obtained the frequency and direction of affiliative and aggressive events on two resident (from year 2008), two within-dialect (from year 2007) and eight across-dialect translocated individuals (five from year 2007 and three from year 2008). We used Mann-Whitney U-test on both behavioral acts and the distance to roosting sites to test for differences between resident and translocated individuals.

## Results

### Vocal Behavior

**Figure 2:**
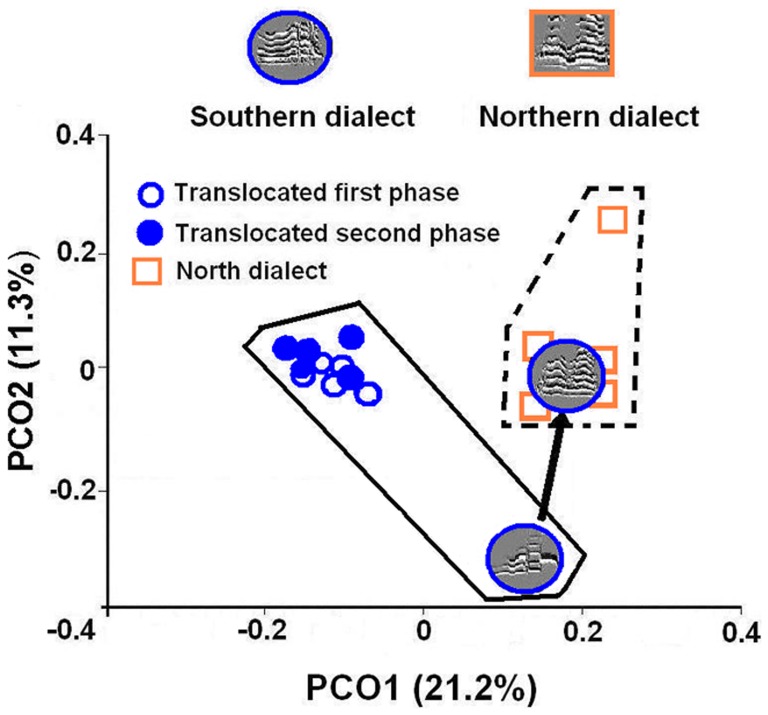
Plot of PCO from contour cross-correlations for calls of yellow-naped amazons. Dashed line delimitates Northern dialects sound space, solid lines delimitate Southern dialect sound space. Points for the vocal imitating individual are illustrated with representative spectrograms, and movement between phases indicated by an arrow. Spectrograms on top show examples of contact calls of residents of the Northern dialect (squared) and translocated individuals from the Southern dialect (rounded).

We found evidence of vocal imitation in the translocated juvenile from the Southern dialect included in the acoustic analysis. This juvenile produced Southern dialect contact calls during the first sampling phase, but had altered its call to match the Northern dialect by the second phase. The contact calls of this imitating individual were more similar to the calls from its release site in the second phase than to its own contact calls recorded during the first phase (Fig. 2). In contrast, all five cross-dialect translocated adults for which we had sufficient samples for analysis retained the structure of their original Southern dialect calls across both phases (Fig. 2). Although all six individuals exhibited some change in the acoustic properties of their contact calls between the two phases, the imitating individual exhibited the largest amount of change in the structure of its contact calls between phases in sound space (Euclidian distances: vocal imitation: 0.337, non vocal-imitation: 0.050±0.010). The discriminant function analysis based on acoustic parameters produced 100% correct classification of calls to Northern or Southern dialect categories based on classification criteria. Although vocalizations from the juvenile were slightly different from those of adults in the first phase (Fig. 2), this analysis classified them as Southern dialect contact calls. In addition, vocalizations from the translocated juvenile after vocal imitation were classified in the Northern dialect after vocal imitation.

### Social Integration

**Figure 3:**
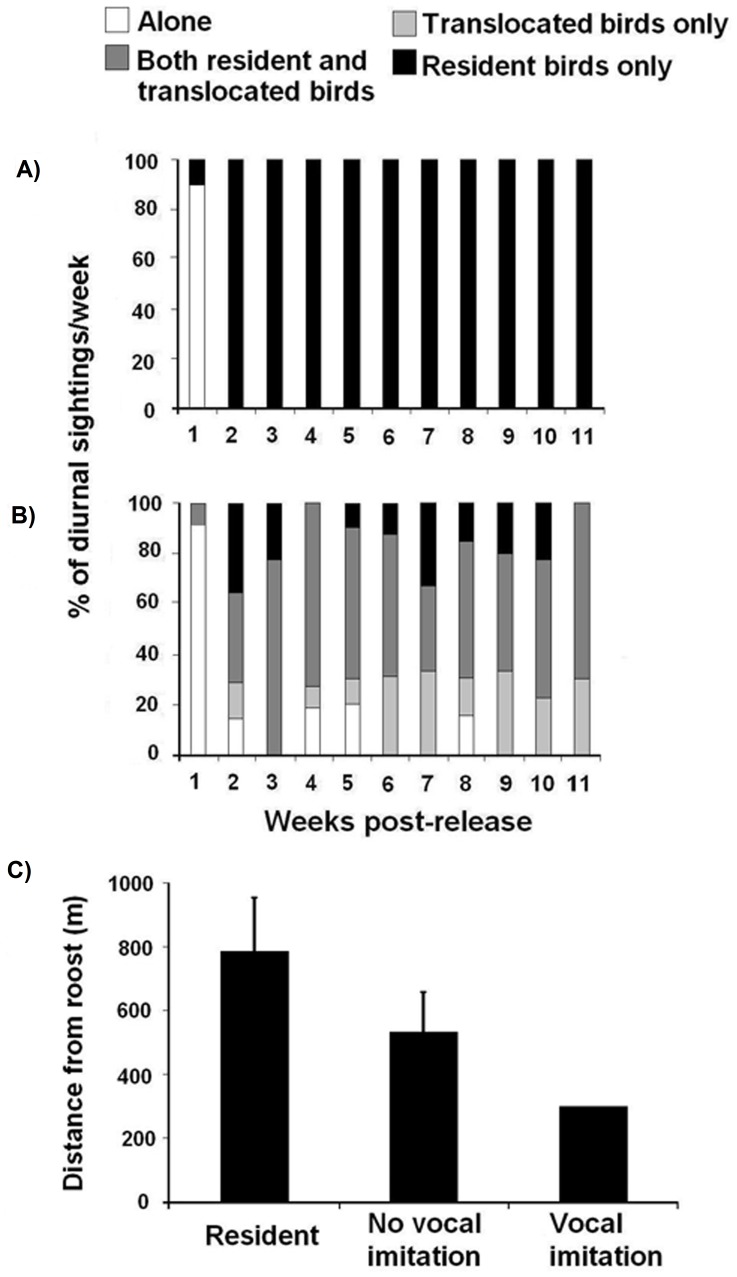
Plot of social association in flocks of a) vocal imitating translocated individuals and b) non-vocal imitating. Note vocal imitation recorded in week six, well after the individual integrated to resident flocks. c) Plot of mean distances (mean ± SE) from sleeping locations to the closest traditional roosting site for residents and translocated birds.

The imitating translocated juvenile completely integrated into resident flocks with exclusively Northern dialect birds by the second week post-release (Fig. 3a), and remained in the resident flocks until the transmitter battery expired 11 months later. Vocal imitation occurred by week six of the monitoring period. By comparison, the non-imitating across-dialect translocated adults were sighted in groups containing other translocated individuals 8 to 78 percent of the time, even though these other translocated bids were always released at either a different site or at a different time (Fig. 3b). Distance from traditional roosts were similar between resident and across-dialect translocated individuals (U_6,8_ = 22, *P* = 0.852); this pattern was observed regardless of whether or not they imitated local calls (Fig. 3c). Four out of the eight individuals translocated within the Northern dialect from Los Inocentes (Table 1) were tracked back to their original trapping site (mean ± SE) 15.5±4.0 days after translocation, while one stayed at the translocation site. Although two of these individuals returning home corresponded to one of the instances in which two individuals were captured at the same time, each individual made its way back home independently with 29 days of separation. We were unable to ascertain the fate of the other individuals as we were unable to acquire their transmitter’s signal at either site. Four out of the 15 translocated individuals from the Southern dialect site returned to their original trapping site in the native dialect 120.3±37.9 (range  = 12±135 days) days after translocation; two individuals returned independently in 2007 and two more individuals returned in 2008 independently. We do not know the fate of eight of the translocated individuals, as last time locations were obtained they were in the release site few days after release.

We conducted 98 (n = 2 birds) and 135 (n = 10 birds) focal observational periods for resident and translocated birds respectively. Few direct social interactions were observed, with social behaviors recorded in only 27% of the observational periods. Aggressive acts were observed in only 3% of the observational periods; across-dialect translocated and resident birds did not differ in the number of aggressive acts received (U_2, 10_ = 2.5, *P* = 0.090) or given (U_2, 10_ = 8.0, *P* = 0.672). No differences were observed between translocated and resident birds in either receiving (16% of observational periods, U_2, 10_ = 7.5, *P* = 0.644) or giving affiliative behaviors (10% of observational periods, U_2, 10_ = 7.5, *P* = 0.645).

## Discussion

This study provides evidence consistent with both the vocal imitation and reduced dispersal hypotheses for dialect maintenance. Both hypotheses have historically been posed as competing explanations for the stability of vocal dialects. We present the first experimental study suggesting that these mechanisms could act in concert for dialect maintenance and further that each mechanism may be age-class related.

This study provides direct evidence consistent with the vocal imitation hypothesis for dialect maintenance. We found that contact calls of the one translocated juvenile closely matched the acoustic characteristics of its new dialect by week six post-release. These altered calls closely resembled the contact calls from the recipient site, Los Ahogados. However, good quality recordings for comparison between phases were recorded in only five tracking sessions (Table 1); hence, the observed timing may not accurately represent the pace of vocal learning due to limited sampling. Despite the lack of information on timing of dispersal for this species, this observation suggests vocal imitation post-dispersal may be a mechanism maintaining dialects by juveniles, the life-history phase at which dispersal is most common [24]. Theoretical models of dialect maintenance have emphasized the importance of vocal learning for dialect stability [25], and vocal imitation has been reported in captive birds moved to new social groups [26], [27]. Although vocal imitation has been commonly invoked to explain the persistence of vocal dialects [15], [28], [29], to our knowledge, this is the first study to empirically demonstrate the imitation of vocal dialects by tracking the vocal and social behavior of individuals experimentally transferred between naturally-occurring vocal dialects.

We also found evidence supporting the idea that reduced dispersal and philopatry in adults could limit the movement across dialect boundaries. None of the adult yellow-naped amazon for which we collected vocal data imitated the local call type. While these translocated adults remained in the foreign dialect, they showed a preference for flocking with other cross-dialect translocated individuals, resulting in segregation from resident flocks (but not roosts). Some of these translocated individuals returned to the capture site 30 km away in their original dialect, a significant distance given observed foraging distances in the species. These patterns suggests that adult yellow-naped amazons are highly philopatric and either unable or unwilling to learn the calls of a new dialect. It is also consistent with the idea that individuals that disperse across dialect boundaries might suffer from reduced fitness due to the lack of call sharing with local individuals; however, it is important to note we did not directly measure the relative fitness of translocated birds. A high degree of philopatry suggests that movement by adults across dialects is limited, as predicted by the reduced dispersal hypothesis. Such philopatry may be driven by natal habitats preference [30] or by social associations.

Recent population level processes in the yellow-naped amazon could be affecting the relative contribution of each of the mechanisms in maintaining vocal dialects in this species. Although indirect estimations of dispersal in the yellow-naped amazon point to considerable flow of individuals across dialect boundaries [12], [16], the low proportion of juveniles captured in our study suggests that high poaching levels may limit actual recruitment of juveniles into the population [23]. Previously reported evaluations of gene flow in this parrot species [12], [16] might not accurately reflect contemporary movement of recruits but rather historical process. Historically, before poaching reached critical levels, dialects may have been maintained more commonly by post-dispersal vocal imitation by juveniles while reduced vocal imitation and higher philopatry of adults contributed less to dialect maintenance. The differences of relative contribution of each mechanism to dialect maintenance may continue to grow due to carry-over effects of poaching. Alternatively, the differences observed in age classes of captured individuals could result from bias in our trapping; hence, reducing our estimation of the current contribution of juveniles to dialect maintenance. We used contact calls and duets for capturing individuals, and adults could have had a stronger response to these calls, biasing capture success towards this age class.

The adaptive value of learning local calls by immigrant birds is uncertain. The password hypothesis, which suggests that imitation of a group’s call is an honest signal of local experience that allows an immigrant to gain group benefits [31], is typically invoked in such situations, albeit with little empirical support. Our evidence does not support the password function for vocal imitation in the yellow-naped amazon. Translocated individuals were able to share roosting sites used by resident birds regardless of whether they imitated local calls. Field observations indicate that despite the uncertainty regarding when vocal imitation actually occurred, the one case of vocal imitation did not occur until several weeks after this bird had joined resident flocks. In addition, we recorded no differences in the number of aggressive and affiliative acts directed towards resident and across-dialect translocated birds. These results suggest that rather than acting as a gateway to social integration, vocal imitation by immigrants is driven by the type and quality of social interactions immigrants have with residents. Alternatively, vocal learning may be driven by longer-term processes such as mate selection; in many parrots mated pairs are close and stable associations maintained over several years ([32] T.F. Wright unp. data). Vocal imitation has been shown to be an important component of mate choice in the budgerigars, *Melopsittacus undulatus* [33], [34].

Our results suggest that the occurrence of vocal imitation may be associated with age-specific constraints or costs. The fact that the only vocal-imitating individual in our study was a juvenile suggests that the motivation for vocal learning may be related to the strength of previous social ties and individual’s age. Dispersal typically occurs in juvenile animals [20], [24]. These differences in dispersal propensity may result in part because individuals with the weakest social ties are more likely to disperse [35]. Hence, it may have been less costly for the translocated juvenile to develop new social ties with local individuals and imitate the local call type than return to its natal area as some adults did. These social ties may also imply that using movement patterns of adults as evidence for dispersal may be misleading, as preexisting social relationships could strongly influence their propensity to return to their capture site. Direct studies of naturally occurring patterns of dispersal using telemetry or other methods are needed to address this point.

Although parrots are known for being open-ended learners, data collected from adults in this study would suggest that learning capabilities are reduced or lacking in adults, as found in other species previously thought to be true open-ended learners [36]. However, vocal imitation is common in captive adults in a wide range of parrot species [37], [38], although individuals may not always exhibit learning due to lack of adequate models or proper motivation [39]. Further experimental evaluations are required to determine whether the limited adult learning observed here is due to fitness costs, physiological constraints or motivation.

## Supporting Information

Sound Example S1 **Sound file: Example of Northern dialect vocalizations at the recipient site.**
(AIF)

Sound Example S2 **Sound file: Example of Southern dialect vocalizations at the source site of translocated birds.**
(AIF)

Sound Example S3 **Sound file: Example of original Southern dialect vocalization of the translocated juvenile in the 1st phase prior to altering vocalization.**
(AIF)

Sound Example S4 **Sound file: Example of Northern dialect vocal imitation by the translocated juvenile in phase two after altering vocalization.**
(AIF)
